# Epidemics past and Present

**Published:** 1890-02-15

**Authors:** 


### The Hospital

A Weekly Journal of

Science, Medicine, Nursing, and Philanthropy.

*For Hospitals, Asylums, Medical Practitioners, Students, Nurses, Families, Parishes, Congregations, and the Charitable Public.*

Vol. VII.—No. 177.] [February 15, 1890.

### EPIDEMICS PAST AND PRESENT.

To those students of human nature who are always painfully conscious of limited knowledge, and who constantly strive to increase the stock of their infor mation and to improve their judgment, the history of disease epidemics is peculiarly interesting and instructive. The illustrious German physician, Professor Hecker, of Frederick William's University at Berlin, maintained the opinion half a century ago "that great epidemics are epochs of development, wherein the mental energies of mankind are exerted in every direction."

No physician now doubts that Europe, including England, has been visited by a genuine epidemic of Influenza of a decidedly severe character. It is said that in previous times an epidemic of influenza has been the precursor and herald of a general outbreak of cholera. It seems therefore well at the present time to consider the whole subject of epidemics from the two standpoints of their influence upon the human race and the feasibility of their prevention. For whatever be the developmental value of a deadly plague, there can be no doubt that most persons will think the progress of the race too dearly purchased at the cost of the lives of a large proportion of the population. If the progressive development of man be the subject of such fixed laws that nothing whatever can prevent its accomplishment, the natural instinct of self-preservation within us all will impel us to look in new directions for developmental stimulus, and by every conceivable means to strive to avoid the deadly whip of general and fatal disease.

The records of past epidemics which can lay any claim to historic value are of comparatively recent date. What was remembered of the "Black Death" has impressed the imagination of European peoples more than any other pestilence of which anything is known. That direful scourge of the fourteenth century desolated Europe, Asia, and Africa alike. It was held to be of Eastern origin, and was so alarming in its outward manifestations and so fatal in its effects that all other epidemics sink into insignificance when compared with it. In those attacked by the disease certain glands of the body, particularly those in the groins and armpits, enlarged and inflamed until they appeared like huge carbuncles. In many cases "black spots" broke out in various parts of the skin, sometimes remaining single and at other times uniting to form large patches of alarming appearance and fatal import. Many of those attacked became stupefied and fell into a deep sleep, losing their speech from palsy of the tongue; others remained sleepless and without rest. The throat and tongue were black, and as if gorged with putrescent blood. "No beverage could assuage the burning thirst of the victims, so that their sufferings continued without alleviation until terminated by death, which many in their despair accelerated with their own hands."

This terrible pestilence spread all over Europe, and in some places as many as a quarter of the population were destroyed. Strangely enough, China was then believed to have been the source and origin of the evil. Just as in these times some have held that the present influenza outbreak is due to the fine dust, laden with microbes, carried by the wind from the drought dried river deposits of China, so in the fourteenth century German accounts say expressly that a "thick stinking mist advanced from the East and spread itself all over Italy." To that atmospheric impurity many of the observers of the time attributed the "Black Death." Whatever degree of soundness there may have been in the theory that the dried and poisonous deposits of Chinese floods gave rise to the Plague, there can be no doubt whatever that the concentrated filth of the large cities of the two continents had their full share in the poisoning of the atmosphere, and of the water which everybody drank. Let the original cause have been what it may, it could only have acted as a lighted match upon trains of favourably disposed gunpowder. The complete indifference of the Middle Ages to sanitation, the poisoning of the wells, the fœtid odours of the streets, the discharging of every kind of unnamable impurity into the streams and rivers, the dirt, the idleness, the ignorance, and the vice of the people all combined to bring about a condition of things in which health was impossible, and the blood of the people, poisoned with the pestilential air of ages, only awaited the contact of the plague microbe to ensure putrescence and death. It is stated that in the East nearly twenty-four millions fell victims to the scourge. In London at least 100,000 persons, more than a quarter of the then population, died. In one burial ground alone upwards of 50,000 corpses, arranged in layers, are said to have been interred. It was currently reported at the time that, taking England throughout, nine persons in every ten perished. That, however, is evidently an exaggeration. "Morals," says the grim chronicler, were "deteriorated everywhere; and when tranquillity was restored the great increase of lawyers, to whom the endless disputes regarding inheritances offered a rich harvest, was astonishing."

Immediately following upon the subsidence of the "Black Death" there broke out the peculiar epidemic known as the "Dancing Mania." That disease was chiefly interesting to physicians and psychologists. It was a remarkable example of the extraordinary mental aberration of which human creatures are capable when brought under the influence of adequate excitement. Very few cases proved fatal, though the exhausting effects of the remarkable contortions to which the victims of "Dancing Mania" subjected themselves must often have brought them to the very verge of death. Medical science as we know it would have stamped out the epidemic in a fortnight.

The "Sweating Sickness" visited England as a widespread epidemic towards the end of the fifteenth century, and produced universal terror. It appears to have been a violent fever, which after a short "rigor" produced in the patient a prostration as sudden as it was complete. There was severe headache and lethargic stupor, with painful oppression of the stomach. The whole body was suffused with a fœtid perspiration. All this took place in the course of a few hours, and the crisis was generally over within the space of a day and a night. The internal heat which the patient suffered was intolerable, yet every refrigerant was certain death. The disease, for the most part, marked for its victims robust and vigorous men. "Two Lord Mayors," says the chronicler, "and six aldermen died within one week, having scarcely laid aside their festive robes" after the coronation of the King. In some respects the sweating sickness resembled the present epidemic.

Space does not permit of a more extended discussion of the subject. From the point of view of the man who regards human affairs as if he himself had little or no personal concern with them, a deadly epidemic may be interesting on two grounds. It clears away the diseased and the weak, and leaves fuller scope for the activities of the robust. Succeeding generations, being descended almost exclusively from the strong survivors of the plague, are not only themselves of greatly increased robustness, but in turn become the progenitors of a bolder and more vigorous offspring. The race thus secures, so to speak, a fresh start, and there is a permanent improvement in the type. That, from the developmental philosopher's point of view, is an undoubted gain.

But those of us who are now living will certainly feel very much inclined to think that our first business is to take care of ourselves and to leave development to take care of itself. If it be the case that epidemic influenza is generally the precursor of epidemic cholera, what can we in Great Britain, with all our modern science and practical skill, do to keep the advancing enemy at bay? There are two things which we may and ought to do; we can strengthen ourselves and improve our surroundings. London can clear away its deadly pall of smoke-laden fog, and it can purify its still more deadly river. Other large towns may walk in the same footsteps. Whether either London or the provinces will do these things is another question. If they do not, then in spite of the improved sanitation of streets and houses an epidemic of cholera would in all probability carry off its victims by tens of thousands. But whatever towns and public bodies may do in their corporate capacity, individuals may train themselves to resist epidemic influences exactly as athletes train themselves to resist fatigue, and swimmers to endure for hours the chilling cold of river or of sea. Is it necessary to say that sober and virtuous habits, regular and nutritious feeding, adequate daily exercise in the open air, moderate expenditure, and peace of mind, combine to bring about a state of health which may almost bid defiance to disease? The knowledge of all these things is now common property; how many in every thousand put that knowledge into practice?

